# Supplementary material for: User-Friendly Replication-Competent MAdV-1 Vector System with a Cloning Capacity of 3.3 Kilobases
Source: Viruses. 2024 May 11;16(5):761. doi: 10.3390/v16050761 (PMC11126015; doi:10.3390/v16050761)
Supplement: Supplementary file 1 [file viruses-16-00761-s001.zip › viruses-2943616-supplementary.pdf]

Table S1. Summary information of PCR primers.

Fragment	Primer name	Sequence	Template	Product (bp)
Kan-Ori	1812KMAdV-F	gccatttttgctaactgtatattattgatgatgtttaaacgatcccgagcggt	pKFAV4GFP	2254
	2208KAN-RsrIIp2	ctgatagcggtctgccacacccagccggcca		
	2208KAN-RsrIIp3	ctgggtgtggcagaccgctatcaggacatagcgttg	pKFAV4GFP	315
	1812KMAdV-R	gccatttttgctaactgtatattattgatgatgtttaaactggaacaacactcaacc		
Linker	2208MAV-ERlinker1	gcttttagga acaatggtct gttgcggaccgcatgtatag tttcct	self-anneal	114
	2208MAV-ERlinker2	cggaccgcat gtatagtttccttcaactagttgatttaaa tgggtc		
	2208MAV-ERlinker3	attctcgagaaaattgccacgacccatttaaatcaactagttgaag		
	2208MAV-ERlinker4	ggttaaggtaaacagtagggggggaattctcgagaaaattgccacga		
EcoRI-RsrII	2208MAV-ERFp1	ggaagggtctgtggtagggaactctgtagt gtataagctacctccctct	pKRMAV1	1579
	2208MAV-ERFp2	gtccgcaacagaccattgttcctaaaagc		
SalI-pIX	2211MAdV-IXCG1	cttggctcctgtctttgatactttttg	pKMAV1-ER	2097
	2211MAdV-IXCG1	ccgtaagttatgtaacgtatacatttttattaaacataaagcgcgtg		
CMVp-GFP1	2211MAdV-IXCG3	gtttaataaaaatgtatacgttacataacttacggtaaatggc	pKFAV4GFP	1323
	2211MAdV-IXCG4	cataagcgtgtgtgtatacttagagtccggacttgtacagctc		
pIX-PvuI	2211MAdV-IXCG5	ggactctaagtatacacacacgcttatgcttaataaaggt	pKMAV1-ER	518
	2211MAdV-IXCG6	tccatgcctttccttcaaagctaca		
CMVp-GFP2	2303FAV4S-F2-GFP1	cgcgctttatgtttaataaaaatgcgttacataacttacggtaaatggc	pKFAV4GFP	1310
	2303FAV4S-F2-GFP2	ttagagtccggacttgtacagctc		
G1K	2303MAdV1-1K-1	ctgtacaagtccggactctaacagaagtttatattggcaccatt	pKan-stuf	1032
	2303MAdV1-1K-2	ttaagcataagcgtgtgtgtagtctctagaaatccactgattac		
G2K	2303MAdV1-2K-1	ttattcctttgtaattcactgaaaatgagttgattgagaccaggagt	pKan-stuf	1217
	2303MAdV1-2K-2	ctggccacatgtggctatttgtctgacatctgccactcctg		
G3K	2303MAdV1-3K-1	ttattcctttgtaattcactgaaatcaaccattcccatactgcc	pKan-stuf	2078
	2303MAdV1-3K-2	ctggccacatgtggctatttatgtggtggtgactgggttt		
G4K	2303MAdV1-4K-1	ttattcctttgtaattcactgaaacttgtgctcctcaggtttct	pKan-stuf	3099
	2303MAdV1-4K-2	ctggccacatgtggctattttcatccctaaccaccaagac		
G5K	2303MAdV1-5K-1	ttattcctttgtaattcactgaaaacaccaaacaccagtccttca	pKan-stuf	2127
	2303MAdV1-5K-2	gaaacctgaggagcacaagc		
	2303MAdV1-5K-3	gcttgtgctcctcaggtttcctcaaagcatcttcccagtc		2086
	2303MAdV1-5K-4	ctggccacatgtggctatttcaaaggaccaaggaagtgaa		
Luciferase-GFP	2309MAdV1-IXCLG1	cgcgctttatgtttaataaaaatgtatacgttacataacttacggtaaatgg	pKHAV108-LGFP	3040
	2309MAdV1-IXCLG1	taagcataagcgtgtgtgtatacttagagtccggacttgtacagctcgtc		
GFP-frag	2008GFPf	gacaaccactacctgagcaccc	Virus genomic DNA	126
	2008GFPr	cttgtacagctcgtccatgcc		
	2008GFPprobe	HEX-tccgccctga gcaaagaccccaac-BHQ1		

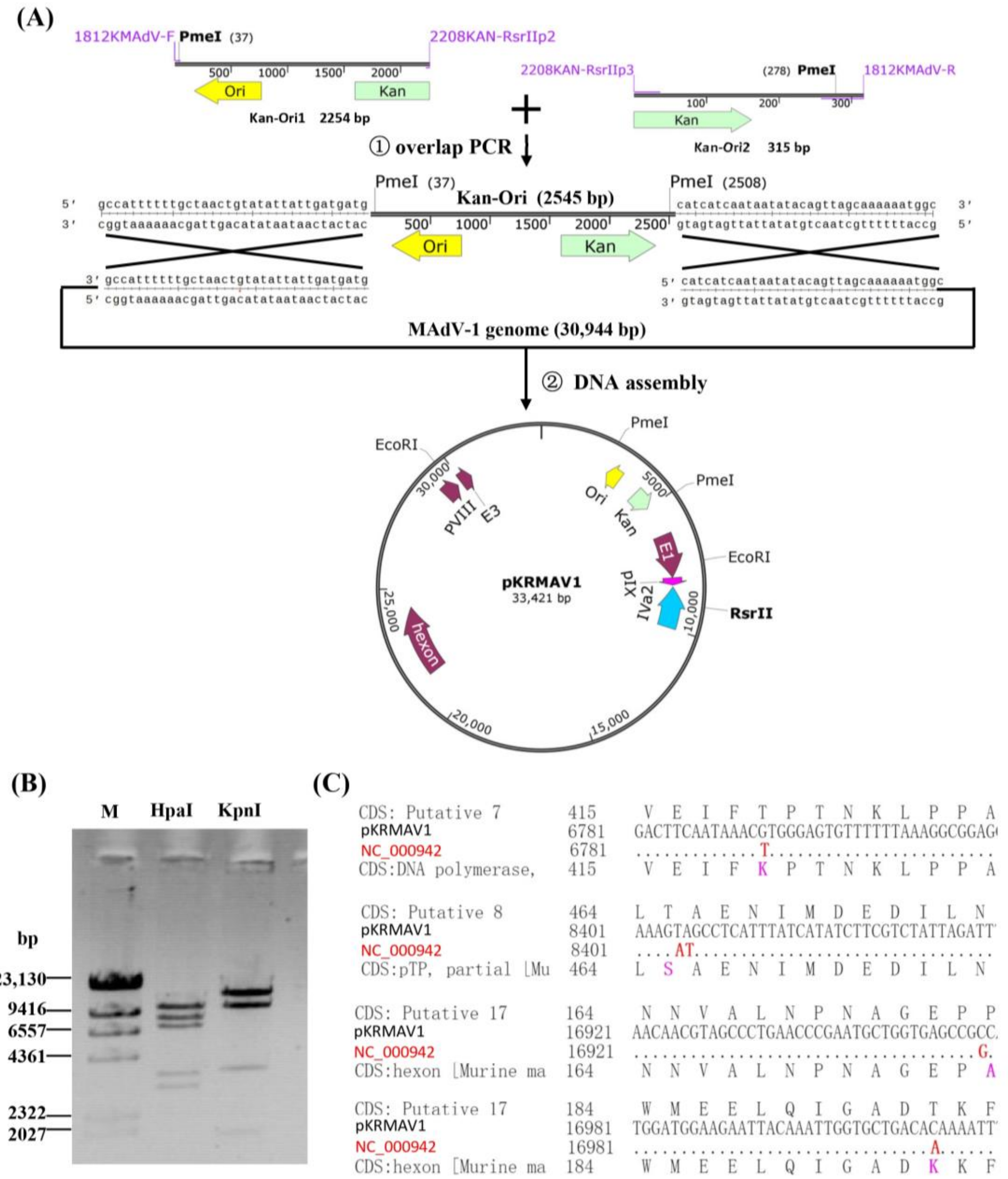

Figure S1. Construction and identification of the infectious plasmid of pKRMAV1.

(A) Kan-Ori fragments were amplified by overlap extension PCR, and a synonymous mutation was introduced to remove the RsrII site in the original template. The MAdV-1 genome was combined with PCR product and subjected to Gibson assembly reaction to generate plasmid pKRMAV1.

(B) Identification of plasmid pKRMAV1 by restriction analysis. DNA was digested with indicated restriction enzymes and resolved on 0.7% agarose gel by electrophoresis. The predicted molecular weights (bp) of digested fragments of pKRMAV1 plasmid were 3068, 3540, 7224, 8487, and 10,601 for HpaI and 1112, 1875, 3692, 10,735, and 15,150 for KpnI. Molecular weights of fragments less than 1000 bp were not given.

(C) Second-generation sequencing of pKRMAV1 plasmid. Five-point mutations were found in the MAdV-1 genome cloned in pKRMAV1 when being aligned to the GenBank sequence of NC_000942.

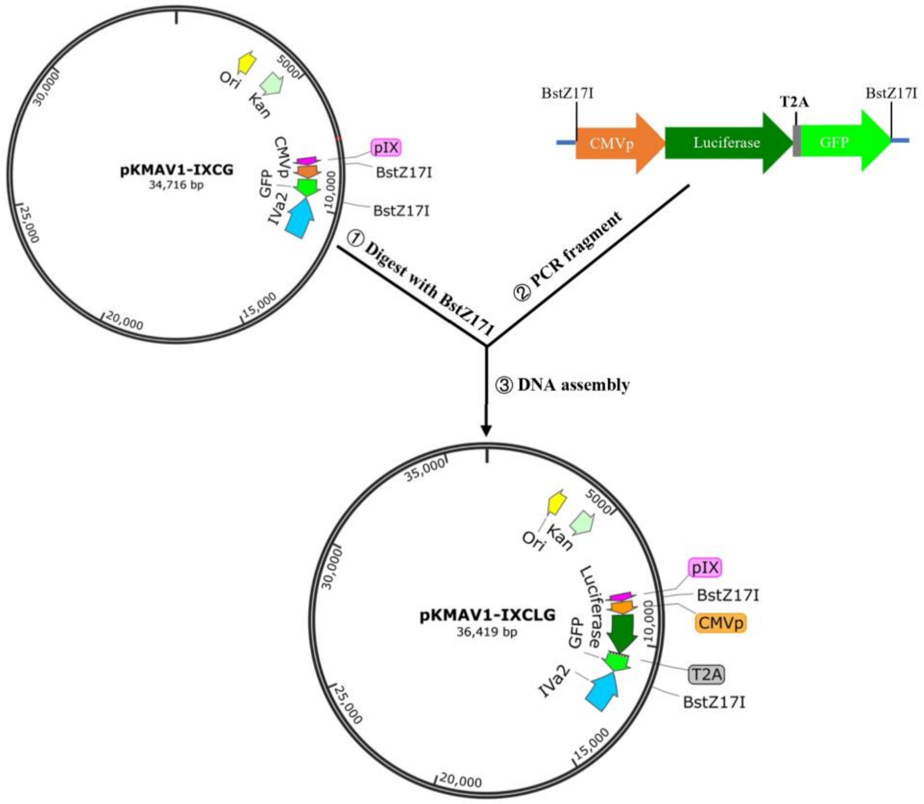

Figure S2. Schematic diagram of constructing pKMAV1-IXCLG plasmid. MAdV-1 adenoviral plasmid pKMAV1-IXCG was digested with restriction enzyme BstZ17I to remove the original exogenous gene expression cassette. A fragment containing CMV promoter, Fluc (firefly luciferase) gene, and GFP gene were amplified by PCR and DNA assembly was carried out to fuse the two fragments to generate plasmid pKMAV1-IXCLG. Of note is that the Fluc coding sequence was linked to that of GFP through the Thosea asigna virus 2A peptide (T2A) coding sequence.

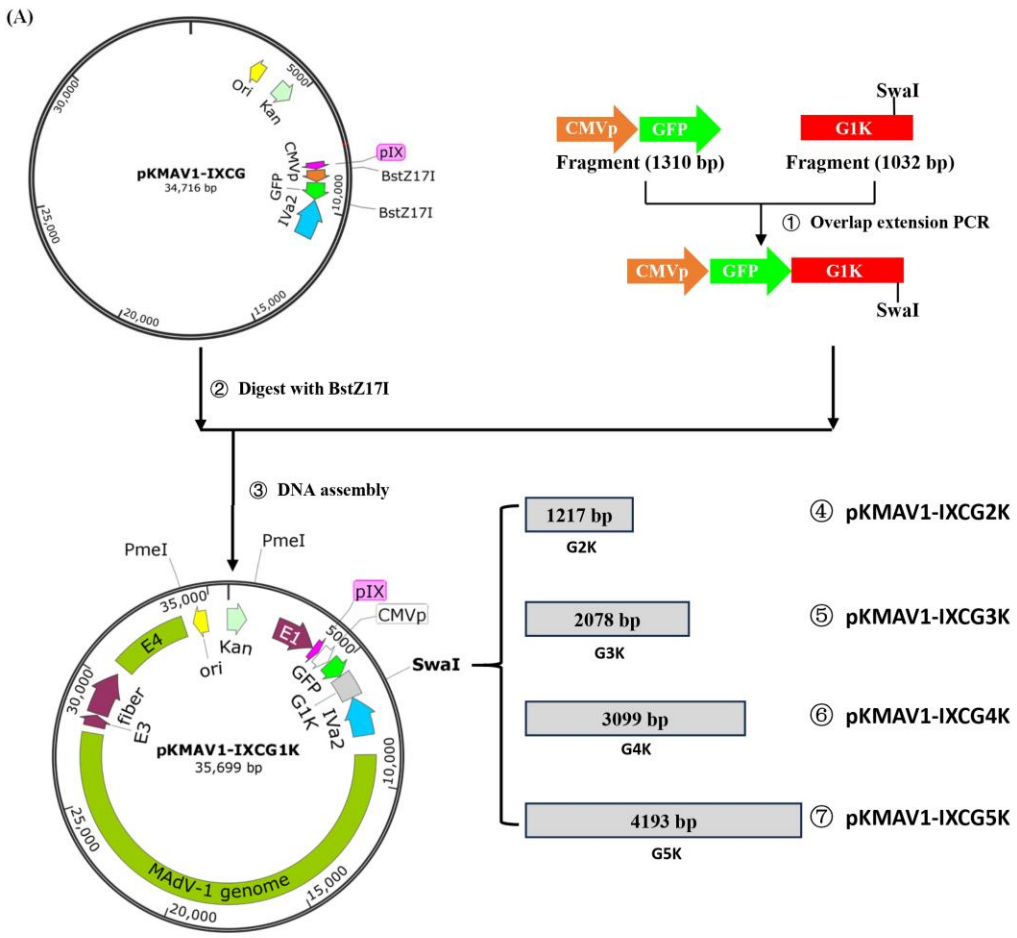

Figure S3. Construction of serial MAdV-1 adenoviral plasmids carrying stuffer DNA in gradually increasing length. CMVp-GFP2 and stuffer DNA of G1K were amplified by PCR and fused to a single fragment by overlap extension PCR, which was integrated between BstZ17I sites in pKMAV1-IXCG to generate pKMAV1-IXCG1K by restriction-assembly. Stuffer DNA fragments of G2K, G3K, G4K, and G5K were amplified by PCR, respectively, and inserted to the SwaI site on the G1K region in pKMAV1-IXCG1K plasmid to generate plasmids of pKMAV1-IXCG2K, pKMAV1-IXCG3K, pKMAV1-IXCG4K, and pKMAV1-IXCG5K by restriction-assembly.

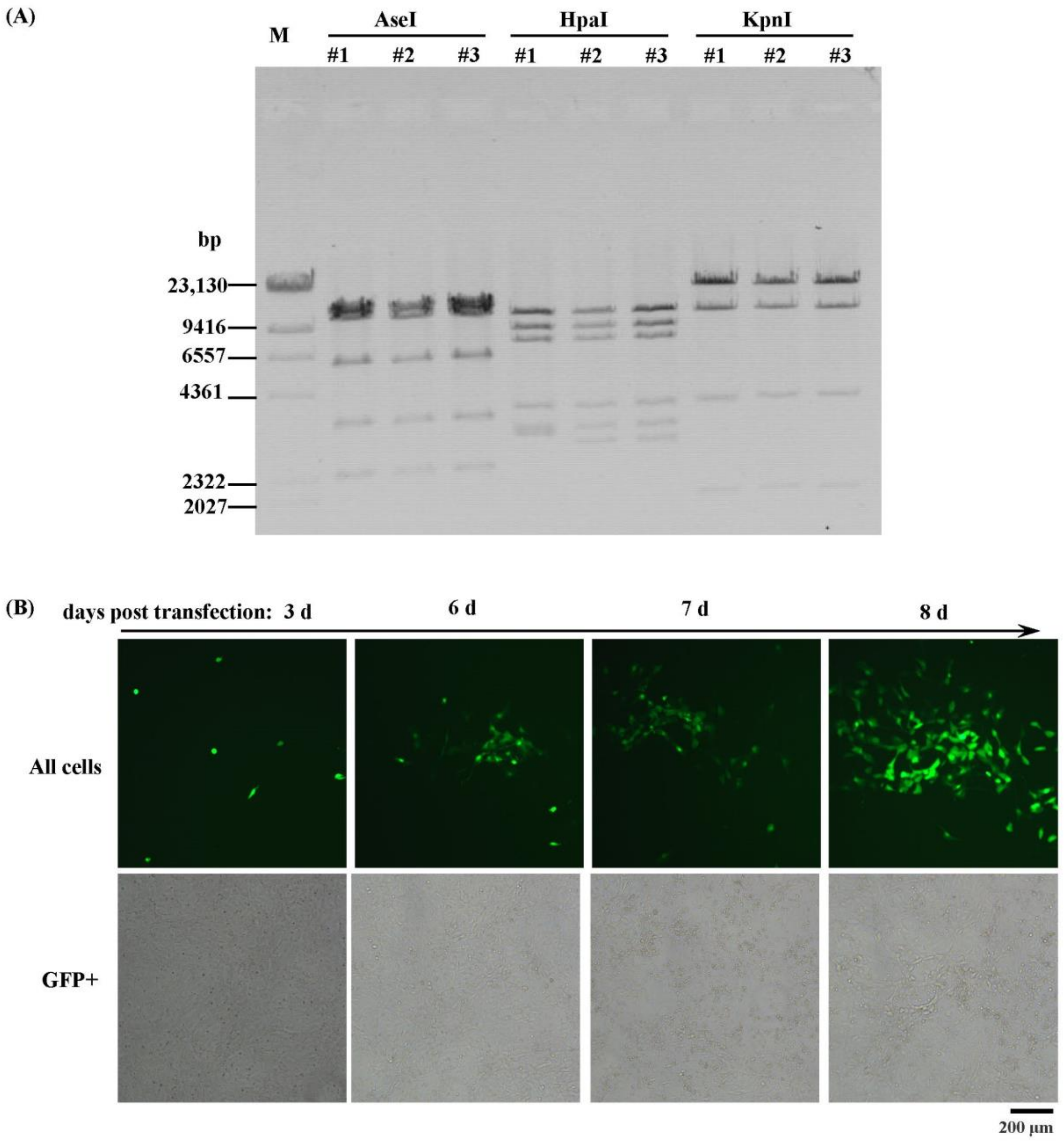

Figure S4. Rescue of recombinant MAdV-1 virus carrying double reporter genes of Fluc and GFP. (A) Identification of pKMAV1-IXCLG plasmid #1-#3 by restriction analysis. DNA was digested with the indicated restriction enzymes and resolved on 0.7% agarose gel by electrophoresis. The predicted molecular weights (bp) of digested fragments of pKMAV1-IXLG plasmid were 1102, 2354, 3381, 5818, 11,085, and 12,382 for AseI; 2930, 3068, 3608, 7224, 8487, and 10,601 for HpaI; and 1112, 1875, 3692, 10,735, and 18,148 for KpnI. Molecular weights of fragments less than 1000 bp were not given.

(B) Rescue of MAdV1-IXCLG virus in PmeI-linearized pKMAV1-IXCLG-transfected NIH/3T3 cells. Foci formed by GFP-positive cells were observed under a fluorescence microscope, suggesting successful virus rescue.

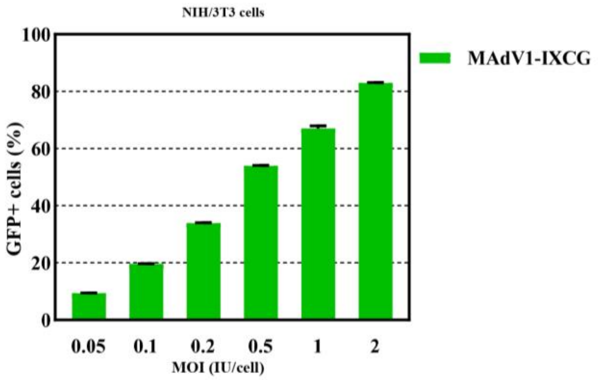

Figure S5. Transduction of MAdV1-IXCG to NIH/3T3 cells. Mouse NIH/3T3 cells were infected with MAdV1-IXCG for 4 hours at various MOIs; the cells were harvested 24 hours post-infection, and the expression of GFP was assayed by flow cytometry.

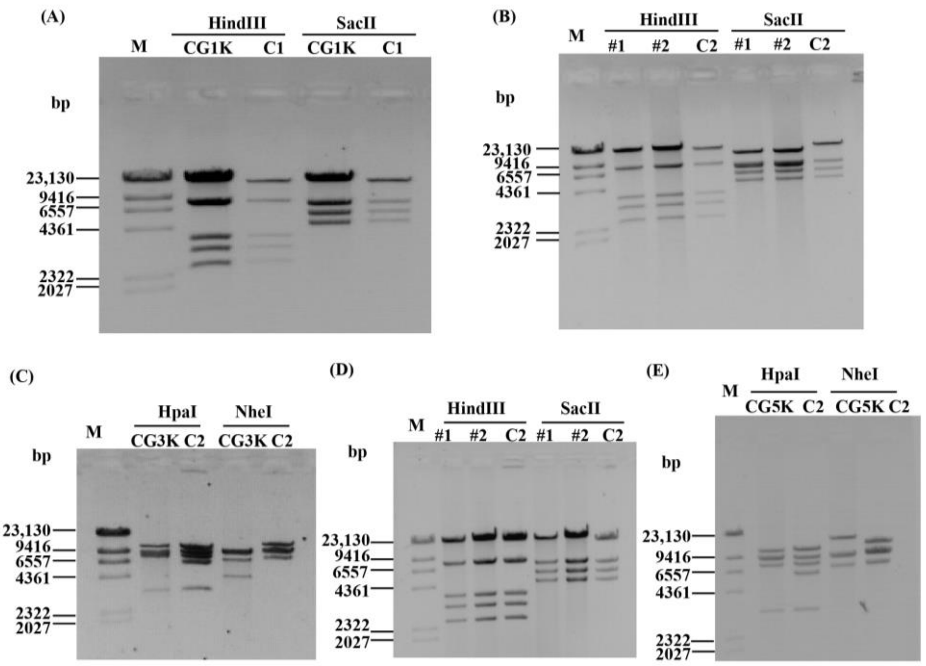

Figure S6. Identification of adenoviral plasmids carrying stuffer DNA by restriction analysis.

(A) Restriction analysis of plasmid pKMAV1-IXCG1K. The predicted molecular weights (bp) of digested fragments of pKMAV1-IXCG1K plasmid were 2484, 3066, 3655, 7813, and 18,681 for HindIII and 4606, 5640, 7268, and 18,185 for SacII. The predicted molecular weights (bp) of digested fragments of pKMAV1-IXCG plasmid were 2484, 3066, 3655, 7813, and 17,698 for HindIII and 4606, 5640, 7268, and 17,202 for SacII.

(B) Restriction analysis of plasmid pKMAV1-IXCG2K #1-#2. The predicted molecular weights (bp) of digested fragments of pKMAV1-IXCG2K plasmid were 2484, 3066, 3655, 7813, and 19,851 for HindIII and 4606, 5640, 6850, 7268, and 12,505 for SacII. The predicted molecular weights (bp) of digested fragments of pKMAV1-IXCG1K plasmid were 2484, 3066, 3655, 7813, and 18,681 for HindIII and 4606, 5640, 7268, and 18,185 for SacII.

(C) Restriction analysis of plasmid pKMAV1-IXCG3K. The predicted molecular weights (bp) of digested fragments of pKMAV1-IXCG3K plasmid were 3068, 7224, 7848, 8487, and 10,601 for HpaI and 4231, 6627, 8467, 8845, and 9289 for NheI. The predicted molecular weights (bp) of digested fragments of pKMAV1-IXCG1K plasmid 3068, 5818, 7224, 8487, and 10,601 for HpaI and 6627, 8467, 8845, and 11,490 for NheI. Molecular weights of fragments less than 1000 bp were not given.

(D) Restriction analysis of plasmid pKMAV1-IXCG4K #1-#2. The predicted molecular weights (bp) of digested fragments of pKMAV1-IXCG4K plasmid were 2484, 3066, 3655, 7813, and 21,732 for HindIII and 4606, 5640, 7268, and 21,236 for SacII. The predicted molecular weights (bp) of digested fragments of pKMAV1-IXCG1K plasmid were 2484, 3066, 3655, 7813, and 18,681 for HindIII and 4606, 5640, 7268, and 18,185 for SacII.

(E) Restriction analysis of plasmid pKMAV1-IXCG5K. The predicted molecular weights (bp) of pKMAV1-IXCG5K plasmid were 3068, 7224, 8487, 9964, and 10,601 for HpaI and 6627, 8467, 8845, and 15,636 for NheI. The predicted molecular weights (bp) of pKMAV1-IXCG1K plasmid were 3068, 5818, 7224, 8487, and 10,601 for HpaI and 6627, 8467, 8845, and 11,490 for NheI. Molecular weights of fragments less than 1000 bp were not given.

C1—control plasmid pKMAV1-IXCG; C2—control plasmid pKMAV1-IXCG1K.

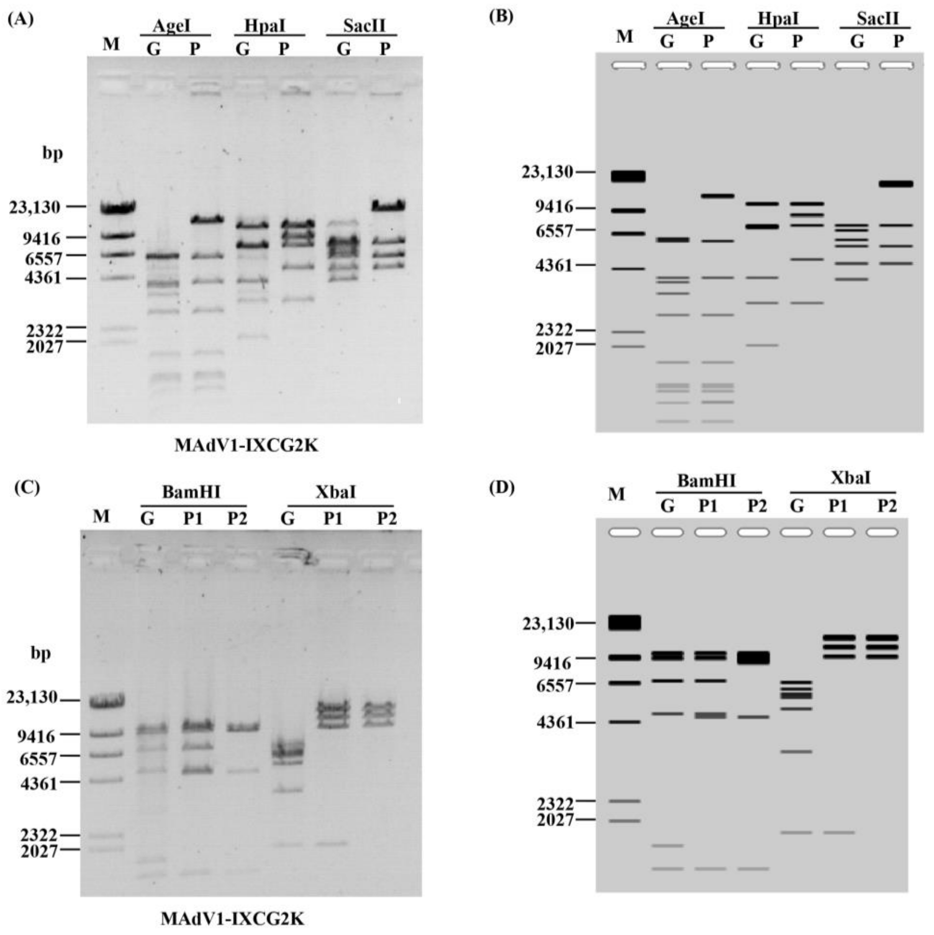

Figure S7. Identification of MAdV1-IXCG2K by restriction analysis of the genomic DNA.

(A) Virus genomic DNA (G) was digested with the indicated restriction enzymes and resolved on 0.7% agarose gel by electrophoresis, and purified DNA of the adenoviral plasmid pKMAdV1-IXCG (P) served as a control. The predicted molecular weights (bp) of digested fragments of MAdV1-IXCG2K genome (G) were 1181, 1326, 1383, 1410, 1748, 2730, 3358, 3766, 3958, 5951, and 6184 for AgeI, 2057, 3068, 3955, 6988, 7224, and 10,601 for HpaI, and 3887, 4606, 5640, 6143, 6850, and 7268 for SacII. The predicted molecular weights (bp) of digested fragments of pKMAV1-IXCG plasmid (P) were 1181, 1201, 1326, 1383, 1410, 1748, 2730, 3958, 5951, and 12,429 for AgeI, 3068, 4835, 7224, 8487, and 10,601 for HpaI, and 4606, 5640, 7268, and 17,202 for SacII. Molecular weights of fragments less than 1000 bp were not given.

(B) Simulated restriction maps generated by SnapGene software for (A).

(C) Virus genomic DNA was digested with restriction enzymes and resolved on 0.7% agarose gel by electrophoresis. The predicted molecular weights (bp) of digested fragments of MAdV1-IXCG2K genome (G) were 1487, 1727, 4737, 6775, 9268, and 10,043 for BamHI and 1881, 3378, 4942, 5585, 5834, 6115, and 6659 for XbaI. The predicted molecular weights (bp) of digested fragments of pKMAV1-IXCG2K plasmid (P1) were 1487, 4559, 4737, 6775, 9268, and 10,043 for BamHI and 1881, 3378, 5585, 5834, 6115, and 14,076 for XbaI. The predicted molecular weights (bp) of digested fragments of pKMAV1-IXCG plasmid (P2) were 1487, 4559, 9268, 9359, and 10,043for BamHI and 3378, 5585, 5834, 6115, and 13,804 for XbaI. Molecular weights of fragments less than 1000 bp were not given.

(D) Simulated restriction maps generated by SnapGene software for (C).

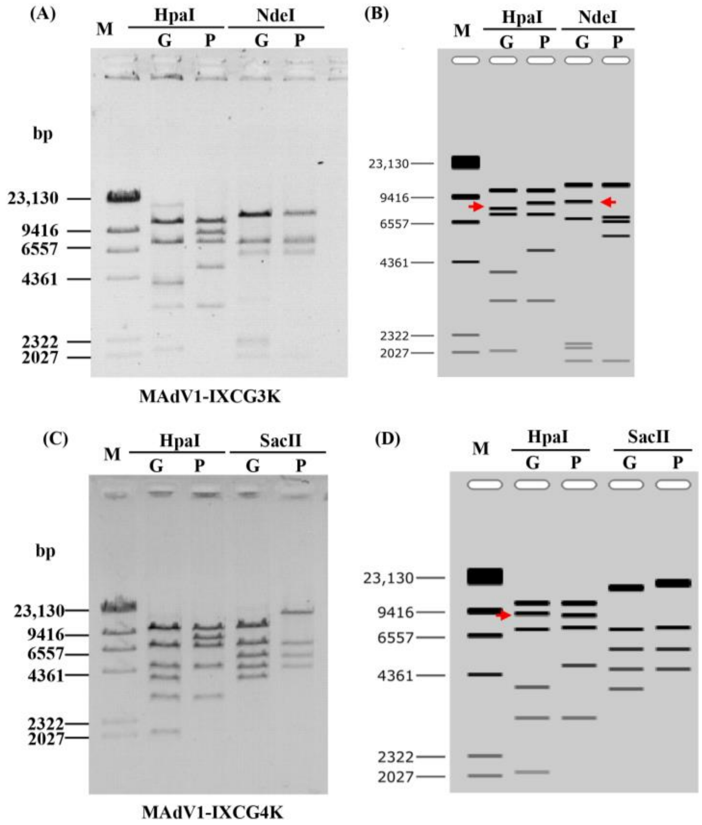

Figure S8. Restriction analysis of viral genomic DNA extracted from purified MAdV1-IXCG3K and MAdV1-IXCG4K viruses. MAdV1-IXCG3K or MAdV1-IXCG4K viruses were subjected to 4 rounds of amplification in NIH/3T3 cells and purified by CsCl ultracentrifugation. Virus genetic DNA was extracted from purified viruses and digested with indicated restriction enzymes.

(A) Restriction analysis of MAdV1-IXCG3K genomic DNA. The predicted molecular weights (bp) of digested fragments of MAdV1-IXCG3K genome (G) were 2058, 3068, 3952, 7224, 7848, and 10,601 for HpaI and 1293, 1908, 2103, 2185, 6997, 8660, and 11,866 for NdeI. The predicted molecular weights (bp) of digested fragments of pKMAV1-IXCG plasmid (P) were 3068, 4835, 7224, 8487, and 10,601 for HpaI and 1297, 1908, 5643, 6765, 6997, and 11,866 for NdeI. Molecular weights of fragments less than 1000 bp were not given.

(B) Simulated restriction maps generated by SnapGene software for (A).

(C) Restriction analysis of MAdV1-IXCG4K genomic DNA. The predicted molecular weights (bp) of digested fragments of MAdV1-IXCG4K genome (G) were 2058, 3068, 3952, 7224, 8869, and 10,601 for HpaI and 3884, 4606, 5640, 7268, and 14,875 for SacII. The predicted molecular weights (bp) of digested fragments of pKMAV1-IXCG plasmid (P) were 3068, 4835, 7224, 8487, and 10,601 for HpaI and 4606, 5640, 7268, and 17,202 for SacII. Molecular weights of fragments less than 1000 bp were not given.

(D) Simulated restriction maps generated by SnapGene software for (C).

The fragments labeled by red arrows were missing in the real agarose gels after electrophoresis, indicating the rearrangement of MAdV1-IXCG3K and MAdV1-IXCG4K genomes.
